# Supplementary material for: Water and chloride as allosteric inhibitors in WNK kinase osmosensing
Source: eLife. 2024 Nov 25;12:RP88224. doi: 10.7554/eLife.88224 (PMC11588334; doi:10.7554/eLife.88224)
Supplement: Supplementary file 1. — (a) Crystallographic data and refinement of WNK1/S382A and WNK1/SA/PEG400. (b) Cell constant superposition comparisons. (c) WNK3/1 expression level, and Activation Loop S308/S382 phosphorylation and activity. (d) WNK1 and WNK3 peptides monitored by liquid chromatography–mass spectrometry (LC–MS). (e) uWNK1 and uWNK3 autophosphorylation assay conditions. (f) Wild-type and E388A uWNK1 kinetic model. (g) Molecular weight versus [WNK] by static light scattering. (h) Crystallographic data and refinement of WNK3/SA/E314A. [file elife-88224-supp1.docx]

**Water and Chloride as allosteric inhibitors in WNK kinase osmosensing**

Liliana R. Teixeira^a^, Radha Akella^a^, John M. Humphreys^a^ , Haixia He^a^, Elizabeth J. Goldsmith^a^

^a^ Department of Biophysics, The University of Texas Southwestern Medical Center, 5323 Harry Hines Boulevard, Dallas, TX 75390-8816, USA.

*Correspondence to: [Elizabeth.Goldsmith@UTSouthwestern.edu](mailto:Elizabeth.Goldsmith@UTSouthwestern.edu)

**This file includes:**

Supplementary files 1a-1h

**Supplementary file 1a**. Crystallographic Data and Refinement of WNK1/S382A and WNK1/SA/PEG400

**WNK1/SA* WNK1/SA/PEG400**

Space group P1 P12_1_1

Unit cell dimensions *a, b, c* (Å) 38.25, 57.72, 65.60 38.32, 56.81, 65.28

Angles α, β, γ (°) 89.0, 89.6, 89.2 90, 95.4, 90

Wavelength (Å) 0.9795 0.9795

Resolution (Å) 50-2.0 (2.0-2.04) 30-2.0 (2.0-2.05)

Unique reflections (last shell) 38697 17297

Completeness (%) (last shell) 89 (83) 96 (93)

I/σ (last shell) 13.3(2.6) 29.8(8.8)

Rsym, Rpim (last shell)^a^ 0.04, 0.045 (0.20, 0.24) 0.14, 0.06 (0.76, 0.29)

Redundancy (last shell) 1.7 (1.1) 7.6 (7.7)

CC1/2 (last shell) 0.99 (0.90) 0.56(0.55)

Wilson B factor 28.9 18.5

**Structure**

Rwork/Rfree^b^  (last shell) 0.16/0.22 (0.21/0.25) 0.199/0.233 (0.20/0.21)

Non-H protein atoms 4900 2281

Waters 453 250

Cryoprotectant (glycerol/PEG400) 9 0

RMSD in bond length (Å)^c^ 0.006 0.016

RMSD in bond angles (°)^c^ 1.38 1.65

Average B-values (Å^2^) 31.9 41.0

Ramachandran plot stats. (%)

Most favored region 94.6 90.8

Disallowed region 1.1 3.4

Molprobity Score 1.9 1.7

Residues missing from the model None 379-385

^a^ R_sym_ = ∑ | I_avg_ - I_j_ | / ∑ I_j_.

^b^ R_factor_ = ∑ | F_o_ - F_c_ | / ∑ F_o_ , where F_o_ and F_c_ are observed and calculated structure factors,

respectively, R_free_ was calculated from a randomly chosen 5% of reflections excluded form

the refinement, and R_factor_ was calculated from the remaining 95% of reflections.

^c^ r.m.s.d is the root-mean-square deviation from ideal geometry.

* Newly crystallized form independent from 6CN9.

**Supplementary file 1b.** Cell constant and Cα-Cα Superposition Comparisons

| A. Coordinate  sets | WNK1/SA  +PEG400 | WNK1/SA  +PEG400 | WNK1/SA  (6CN9) | pWNK1  (5W7T) |
| --- | --- | --- | --- | --- |
| Crystal system | Monoclinic  indexing | Triclinic  indexing | Triclinic | Monoclinic |
| Space Group | P2_1_ | P1 | P1 | P2_1_ |
| *a (*Å)  *b (*Å)  c *(*Å)  *α* (˚)  *β* (˚)  *γ* (˚) | 38.32  56.81  65.28  90.0  95.4  90.0 | 38.32  56.82  65.89  90.1  95.4  90.0 | 38.31  57.77  65.66  91.3  90.0  90.9 | 45.18  62.23  120.90  90.0  92.3  90.0 |
| Cα-Cα overlay | PEG-6CN9A | PEG-6CN9B | PEG-6CN9 | PEG-pWNK |
| *Å* | 0.79 | 1.3 | 3.2 | 0.82 |

**Supplementary file 1c.** WNK3/1 expression level, and activation loop S308/S382 phosphorylation and activity

| **Mutants** | **Expression** | **Purification** | **Phos (%) from**  ***E. coli*** | **Auto-phos** | **Cl^-^ Sensitivity** |
| --- | --- | --- | --- | --- | --- |
| wt WNK3 | Very good | 26 mg/L | 98% | N/A | Very Sensitive |
| WNK3/E314A | Ok | 7 mg/L | 100% | Much faster | Insensitive |
| WNK3/E314Q | Ok | 6 mg/L | 99% | Faster | Insensitive |
| WNK3/K236A | Very good | 20 mg/L | 97% | Similar WT | Similar WT |
| WNK3/K307A | Good | 16 mg/L | 90% | Similar WT | Similar WT |
| WNK3/M301A | Very good | 23 mg/L | 96% | Slower | Very sensitive |
| WNK3/Y346F | Very good | 25 mg/L | 97% | Slower | Very sensitive |
| WNK3/D279N | Ok | 9 mg/L | 92% | Slower | Very sensitive |
| wt WNK1 | Good | 15 mg/L | 95% | Slower | Very Sensitive |
| WNK1/E388A | Ok | 10 mg/L | 94% | Faster WNK1 | < wt WNK1 |

**Supplementary file 1d.** WNK1 and WNK3 peptides monitored by LC-MS

**Monitored peptide (M+2H)^2+^ % acetonitrile**

Wild type WNK1 AKSVIGTPEFMAPEMY 885.5 22.5%

AKS*VIGTPEFMAPEMY 925.5 23.5%

WNK1/E388A AKSVIGTPAFMAPEMY 885.5 18.0%

AKS*VIGTPAFMAPEMY 925.5 18.8%

Wild type WNK3 MRTSF 641.1 10.1%

MRTS*F 721.1 11.5%

AKSVIGTPEFMAPEMY 885.5 22.5%

AKS*VIGTPEFMAPEMY 925.5 23.5%

E314A MRTSF 641.1 10.1%

MRTS*F 721.1 11.5%

AKSVIGTPAFM 561.5 18.0%

AKS*VIGTPAFM 601.5 18.8%

K236A MRTSF 641.1 10.1%

MRTS*F 721.1 11.5%

AKSVIGTPEFMAPEMY 885.5 22.5%

AKS*VIGTPEFMAPEMY 925.5 23.5%

K307A MRTSF 641.1 10.1%

MRTS*F 721.1 11.5%

AASVIGTPEFMAPEMY 856.9 30.3%

AAS*VIGTPEFMAPEMY 897.0 33.2%

M301A ARTSF 581.1 7.3%

ARTS*F 661.1 8.6%

AKSVIGTPEFMAPEMY 885.5 22.5%

AKS*VIGTPEFMAPEMY 925.5 23.5%

Y346F MRTSF 641.1 10.1%

MRTS*F 721.1 11.5%

AKSVIGTPEFMAPEMY 885.5 22.5%

AKS*VIGTPEFMAPEMY 925.5 23.5%

D297N MRTSF 641.1 10.1%

MRTS*F 721.1 11.5%

AKSVIGTPEFMAPEMY 885.5 22.5%

AKS*VIGTPEFMAPEMY 925.5 23.5%

______________________________________________________________________________________

Wild type and mutant WNK1- and WNK3-specific chymotrypsin-derived activation loop peptides monitored

to generate autophosphorlation progress curves. M*/z* for the (M+2H)^2+^ of peptide and percent

acetonitrile where peptide elutes from a C18 HPLC column (0.1% formic acid in acetonitrile/water

HPLC mobile phase).

**Supplementary file 1e.** uWNK1 and uWNK3 autophosphorylation assay conditions

| Component |  |  |  |  |
| --- | --- | --- | --- | --- |
| HEPES pH7.4 | 20 mM | 20 mM | 20 mM |  |
| Mg Gluconate | 20 mM | 20 mM | 20 mM |  |
| Final [Cl^-^] | 50 mM | 150 mM | 250 mM |  |
| ATP | 5 mM | 5 mM | 5 mM |  |
| uWNK1/uWNK3 | 4 μM | 4 μM | 4 μM |  |
| Final Volume | 50 μL | 50 μL | 50 μL |  |
| Guanidine-HCl to final conc. of 1M to stop the reaction.. | | |  |  |

**Supplementary file 1f.** Wildtype and E388A uWNK1 autophosphorylation were fit to a basic autocatalytic mechanism using DynaFit software. Modeled progress curves superimposed on mass spectrometry data are shown in Figure 5E and 5F.

**Mechanism**

uWNK + pWNK → 2 pWNK :k_P_

uWNK + ion ⇄ uWNK_ion :ion_on ion_off

**ODE System**

d[uWNK]/dt = - kP[uWNK][pWNK] - ion_on[uWNK][ion] + ion_off[uWNK_ion]

d[pWNK]/dt = + kP[uWNK][pWNK]

d[ion]/dt = - ion_on[uWNK][ion] + ion_off[uWNK_ion]

d[uWNK_ion]/dt = + ion_on[uWNK][ion] - ion_off[uWNK_ion]

**Optimized Parameters**

**uWNK1 wt uWNK1 E388A**

| Parameter | Initial Value | Final Value | Std. Error | CV(%) |
| --- | --- | --- | --- | --- |
| KP | 0.18 | 0.183 | 0.008 | 4.4 |
| Ion_on | 2e6 | 3.54e^-6^ | 5.4e^-7^ | 15.2 |
| Ion_off | 0.2 | 0.119 | 0.013 | 11.1 |
| Kionbound |  | 2.98e^-5^ |  |  |

| Parameter | Initial Value | Final Value | Std. Error | CV(%) |
| --- | --- | --- | --- | --- |
| KP | 0.18 | 0.150 | 0.007 | 4.7 |
| Ion_on | 2e6 | 4.15e^-6^ | 6e^-7^ | 14.4 |
| Ion_off | 0.2 | 0.103 | 0.011 | 10.5 |
| Kionbound |  | 4.03e^-5^ |  |  |

**Supplementary file 1g.** Molecular weight vs [WNK] by static light scattering

| **WT/ Mutants** | **MW at 0.8 mg/mL (KDa)** | **St. Dev.** | **MW at 1.8 mg/mL** | **St. Dev.** |
| --- | --- | --- | --- | --- |
| wt WNK3 | 65 | 0.6 | 78 | 0.5 |
| WNK3/E314Q | 51 | 0.2 | 52 | 0.1 |
| WNK3/E314A | 56 | 0.1 | 77 | 1.1 |
| WNK3/K236A | 61 | 0.3 | 85 | 0.2 |
| WNK3/K307A | 59 | 0.3 | 65 | 0.3 |
| WNK3/M301A | 64 | 0.4 | 69 | 0.2 |
| WNK3/Y346F | 68 | 0.8 | 76 | 0.1 |
| WNK3/D279N | 39 | 0.1 | 41 | 0.1 |
| wt WNK1 | 38 | 0.5 | 35 | 0.1 |
| WNK1/E388A | 39 | 0.1 | 35 | 0.6 |

**Supplementary file 1h**. Crystallographic Data and Refinement of WNK3/SA/E314A

**WNK3/SA/E314A**

Space group P12_1_1

Unit cell dimensions *a, b, c* (Å) 50.17, 113.60, 67.52

Angles α, β, γ (°) 90, 101.4, 90

Wavelength (Å) 0.9795

Resolution (Å) 43-3.3 (3.36-3.3)

Unique reflections (last shell) 7500

Completeness (%) (last shell) 70(55)

I/σ (last shell) 9.8(1.0)

Rsym, Rpim (last shell)^a^ 0.17, 0.08 (0.76, 0.54)

Redundancy (last shell) 5.2 (3.7)

CC1/2 (last shell) 0.96(0.60)

Wilson B factor 81.9

**Structure**

Rwork/Rfree^b^  (last shell) 0.188/.270 (0.30/0.42)

Non-H protein atoms 4255

Waters 117

RMSD in bond length (Å)^c^ 0.004

RMSD in bond angles (°)^c^ 1.62

Average B-values (Å^2^) 87.1

Ramachandran plot stats. (%)

Most favored region 88.4

Disallowed region 1.6

Molprobity Score 1.9

Residues missing from the model A:/303-314;B:/303-315

^a^ R_sym_ = ∑ | I_avg_ - I_j_ | / ∑ I_j_.

^b^ R_factor_ = ∑ | F_o_ - F_c_ | / ∑ F_o_ , where F_o_ and F_c_ are observed and calculated structure factors,

respectively, R_free_ was calculated from a randomly chosen 5% of reflections excluded form

the refinement, and R_factor_ was calculated from the remaining 95% of reflections.

^c^ r.m.s.d is the root-mean-square deviation from ideal geometry.
